# Proactive Bias Mitigation When Using Online Survey Panels for Self-Reported Use of Illicitly Manufactured Fentanyl in the General Adult Population

**DOI:** 10.1001/jamahealthforum.2025.4011

**Published:** 2025-11-07

**Authors:** Joshua C. Black, Karilynn M. Rockhill, Nicole Schow, Andrew A. Monte

**Affiliations:** 1 Rocky Mountain Poison & Drug Safety, Denver Health and Hospital Authority, Denver, Colorado; 2 University of Colorado School of Medicine, Aurora

## Abstract

**Importance:**

Illicitly manufactured fentanyl remains a public health threat and trustworthy measurements in prevalence are crucial to public health approaches. Low prevalence behaviors, such as route of administration of illicitly manufactured fentanyl, may have shifted over time, which changes community risk profiles.

**Objective:**

To assess the impact of bias mitigation methods in an online survey sample and quantify changes in routes of administration in illicitly manufactured fentanyl use over time.

**Design, Setting, and Participants:**

This repeated cross-sectional survey included US adults 18 years and older in an online, panel-based general population sample fielded twice yearly, in spring and autumn. Corrections for demographic and nondemographic composition bias using calibration weights and removal of misclassification from careless/inattentive responses were applied. Data were collected from April 2022 to October 2024, and data were analyzed in May 2025.

**Main Outcomes and Measures:**

Self-reported use of illicitly manufactured fentanyl in the past 12 months and routes of administration, which included oral, injection, smoking, or snorting. Weighted frequency and percentages were calculated.

**Results:**

In the full 2022-2024 sample of 175 058 respondents where misclassification removal and calibration was applied, 50.6% (95% uncertainty interval [UI], 50.3-60.0) were female, 48.1% (95% UI, 47.8-48.4) were male, and 1.3% (95% UI, 1.2-1.3) were transgender, nonbinary, or something else, and the median (IQR) age was 47 (32-62) years. The bias-mitigated prevalence estimate of illicitly manufactured fentanyl use in the last 12 months increased from 0.7% (95% UI, 0.7-0.8) in 2022 to 1.1% (95% UI, 1.0-1.2) in 2024. Oral use of illicitly manufactured fentanyl increased from 35.9% (95% UI, 31.1-40.7) in 2022 to 44.4% (95% UI, 40.3-48.5) in 2024, which was the most common route of administration. In 2024, use by smoking was 37.9% (95% UI, 34.1-41.6), use by snorting was 27.1% (95% UI, 23.5-30.7), and use by injection was 24.5% (95% UI, 21.3-27.7). Importantly, bias mitigation cumulatively reduced the national estimate of illicitly manufactured fentanyl by 70.9% in 2024 (from 3.9% [95% UI, 3.8-4.1] when neither was applied to 1.1% [95% UI, 1.0-1.2]), an important factor when considering prevalence and change over time.

**Conclusions and Relevance:**

Results of this survey study suggest that fentanyl use has shifted toward oral use, which may contribute to observed lower mortality rates despite an increase in prevalence of use. Methods intended to reduce systematic bias have a strong influence on low prevalence behavior estimates and should be implemented for all survey-based drug use surveillance.

## Key Points

**Question:**

How much does proactive bias mitigation affect prevalence estimates of illicitly manufactured fentanyl use when using an online general population survey?

**Findings:**

In this survey study including 175 058 respondents, the 2024 estimate of illicitly manufactured fentanyl use decreased from 3.9% to 1.1% after bias mitigation, a 70.9% drop. Bias-mitigated estimates of oral use of illicitly manufactured fentanyl increased from 35.9% in 2022 to 44.4% in 2024, which was the most common route of administration.

**Meaning:**

When using online surveys, proactive bias mitigation is essential to produce valid estimates.

## Teaser

This survey study assesses the impact of bias mitigation methods in an online survey sample and quantifies changes in routes of administration in illicitly manufactured fentanyl use over time.

## Introduction

One essential component of multisource public health monitoring is general population surveys (GPSs) that measure population-level drug prevalence, related behaviors, and medical outcomes.^1^ Low prevalence, high-risk behaviors, such as route of administration of illicitly manufactured fentanyl (IMF), are critical aspects of community risk profiles. Federal household-based GPS, like the National Survey on Drug Use and Health, have been in operation for decades. Online, nonprobability GPS have emerged as an important complement to federal surveys, with several advantages. Nonprobability GPS generate estimates more rapidly, cost less, and can be flexibly modified because fielding is shorter.^2,3^ However, the validity of nonprobability GPS is underreported.

Validity of estimates from all studies, including GPS, depend on (ultimately) untestable models, codified by the study design and analysis.^4^ Challenges to all GPS include measuring low-prevalence items, crafting reading level–appropriate material, maintaining respondent engagement, enriching underrepresented groups, and more. Probability GPSs are chiefly limited by selection bias from nonresponse and sampling frame mismatches (eg, household surveys not including unhoused individuals). In nonprobability GPS, nonrepresentative sample compositions can bias estimates if mismatches are correlated with outcomes. Correcting only demographic factors via quota designs or post-hoc weighting is often insufficient to mitigate composition bias in domain-specific estimates.^5^ Careless or inattentive responses, where respondents stop engaging and simply click answers in random and nonrandom fashions,^6,7^ inflate drug use estimates.^2^ The prevalence of respondents answering carelessly may be high with online-based interfaces, where estimates ranged from 3% to 70%.^8^

Recent IMF use estimates among adults in the US range from 0.3% to 7.5%, derived from probability and nonprobability GPS.^3^ IMF use remains a public health threat, and trustworthy prevalence measurements are crucial to public health strategies since biased estimates can lead to ineffective public health responses. The objectives of this article were to assess the impact of bias mitigation methodology in a nonprobability GPS by correcting nondemographic composition mismatches and removing respondents who answered carelessly. We generate bias-corrected estimates of prevalence and routes of administration of IMF in the US.

## Methods

### Data Source and Measures

The Survey of Non-Medical Use of Prescription Drugs is a repeated, cross-sectional nonprobability GPS recruiting adults from an online consumer research panel owned and operated by Kantar’s Profiles division, fielded once in spring and once in autumn. Quota sampling ensures proportional census region distributions and a 50/50 split of biological sex. Two bias mitigation methods relevant to nonprobability GPS were implemented in the Survey of Non-Medical Use of Prescription Drugs and validated in prior work: (1) exclusion of careless responses using 5 detection methods and (2) predefined calibration weighting, including nondemographic, health-related factors.^2,9^ From April 2022 to October 2024, respondents were asked about IMF or IMF analogue use and routes of administration within the last 12 months at the time of the survey. Survey design, question wording, calibration and careless exclusion methods, and fielding details can be found in the eMethods in Supplement 1.^10^ The study was approved by the Colorado Multiple Institutional Review Board. This study followed the Improving the Quality of Web Surveys: the Checklist for Reporting Results of Internet E-Surveys (CHERRIES) reporting guideline, and informed consent to be surveyed was collected via the online form prior to starting the questionnaire.

### Statistical Analysis

Three approaches to estimating IMF use in the past 12 months, route of administration, and demographic and health characteristics were created. First, estimates directly from the sample were calculated, where only biological sex and census region were controlled by quota sampling; no weighting was applied (termed *neither applied*). Second, respondents showing careless patterns were excluded; again, no weighting was applied (termed *misclassification removal applied*). Third, both were applied (termed *misclassification removal and calibration applied*). Uncertainty intervals (UI) were calculated using a bootstrap method (250 repetitions) because sampling was nonprobabilistic. Statistics were calculated with SAS version 9.4 (SAS Institute). Data were analyzed in May 2025.

## Results

**Figure 1.**
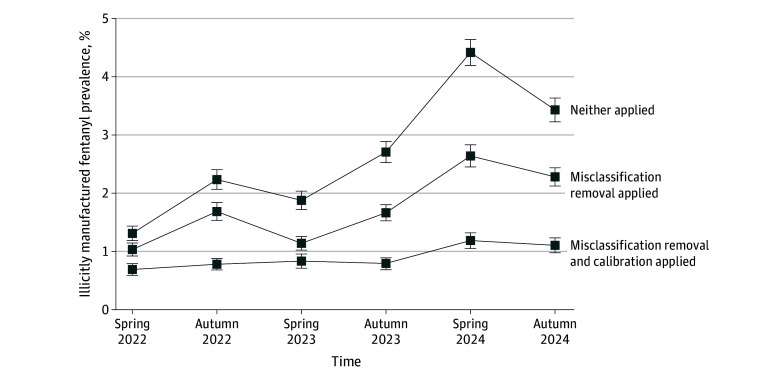
Association of Bias Mitigation With Illicitly Manufactured Fentanyl Estimates in a General Population Survey, 2022-2024 Both approaches to bias mitigation (calibration and misclassification) had noticeable impacts on estimates of prevalence of use of illicitly manufactured fentanyl in the last year among adults in the US. In the neither applied group, only biological sex and census region were controlled by quota sampling; no weighting was applied. In the misclassification removal applied group, respondents showing careless patterns were excluded, with no weighting applied. In the misclassification removal and calibration applied group, both were applied. Error bars indicate 95% uncertainty intervals.

In the full 2022-2024 sample of 175 058 respondents where misclassification removal and calibration was applied, 50.6% (95% UI, 50.3-60.0) were female, 48.1% (95% UI, 47.8-48.4) were male, and 1.3% (95% UI, 1.2-1.3) were transgender, nonbinary, or something else, and the median (IQR) age was 47 (32-62) years. Both bias mitigation approaches substantially reduced IMF prevalence estimates of past-year use (Figure 1), and seasonal variance was observed in unadjusted estimates. Using 2024 data, the annual estimate decreased from 3.9% (95% UI, 3.8-4.1) when neither was applied to 1.1% (95% UI, 1.0-1.2) when both were applied, a 70.9% reduction. Bias-mitigated IMF prevalence was 0.7% (95% UI, 0.7-0.8) in 2022, 0.8% (95% UI, 0.7-0.9) in 2023, and 1.1% (95% UI, 1.0-1.2) in 2024. Careless response exclusions were less than 5% of the final samples (eTable in Supplement 1) but resulted in large reductions in IMF estimates. Bias mitigation also adjusted characteristics of adults who use IMF (Table; full 2024 data). The bias-mitigated subpopulation characteristics increased in percentage of male respondents and increased percentages of both younger (aged 18 to 29 years) and older (55 years and older) respondents. Percentage of respondents with moderate or severe symptoms of anxiety and depression decreased; paradoxically, the percentage of respondents with very high well-being also decreased. Finally, other substance use markers also uniformly decreased with bias mitigation.

**Table.** Demographic and Health Characteristics of Adults Using Illicitly Manufactured Fentanyl in the Past 12 Months With and Without Bias Mitigation, 2024

Characteristic	Adults, % (95% UI)^a^		
	Neither bias mitigation applied	Misclassification removal applied	Misclassification removal and calibration applied
Sample size, No.	2354	1419	1419
Estimated population size, millions	NA^b^	NA^b^	3.04 (2.79-3.28)
Self-reported gender			
Female	32.5 (30.7-34.4)	35.4 (32.9-38.0)	32.4 (28.9-36.0)
Male	66.2 (64.3-68.1)	63.3 (60.7-65.8)	66.5 (63.0-70.1)
Transgender, nonbinary, or something else	1.3 (0.8-1.7)	1.3 (0.7-1.9)	1.0 (0.2-1.9)
Age category, y			
18-29	21.3 (19.6-23.0)	19.4 (17.2-21.5)	30.0 (25.9-34.1)
30-54	74.7 (73.0-76.4)	75.8 (73.5-78.2)	65.3 (61.3-69.2)
≥55	4.0 (3.2-4.8)	4.8 (3.6-6.0)	4.7 (3.1-6.4)
Self-reported race and ethnicity			
Non-Hispanic African American/Black	8.5 (7.5-9.6)	7.8 (6.4-9.1)	8.8 (6.2-11.5)
Hispanic/Latinx	25.2 (23.5-26.9)	22.3 (20.2-24.5)	22.4 (19.0-25.9)
Non-Hispanic White	61.8 (59.8-63.8)	65.0 (62.5-67.4)	62.3 (58.3-66.3)
Other non-Hispanic race^f^	4.4 (3.7-5.2)	4.9 (3.8-6.1)	6.4 (4.3-8.6)
Education			
≤High school	24.0 (22.4-25.6)	26.2 (23.9-28.5)	27.5 (23.6-31.4)
Some college or associate degree	25.4 (23.7-27.1)	26.8 (24.5-29.1)	27.0 (23.5-30.4)
Trade school or bachelor’s degree	32.9 (31.1-34.8)	32.6 (30.4-34.7)	32.0 (27.9-36.1)
≥Graduate degree	17.7 (16.1-19.2)	14.4 (12.7-16.2)	13.5 (10.8-16.3)
Mental health and well-being			
Moderate or severe anxiety symptoms^c^	59.4 (57.5-61.4)	54.8 (52.2-57.3)	54.1 (49.9-58.3)
Moderate or severe depression symptoms^d^	72.3 (70.4-74.3)	66.6 (64.0-69.2)	63.9 (59.9-67.9)
Very high well-being^e^	29.1 (27.2-31.0)	26.1 (23.8-28.5)	24.5 (21.3-27.6)
Substance use in the last 12 mo			
Alcohol used	58.6 (56.6-60.6)	55.2 (52.5-57.9)	55.4 (51.3-59.5)
THC used	51.4 (49.3-53.6)	46.5 (44.0-49.0)	46.6 (42.8-50.4)
Opioid analgesics used	70.2 (68.5-72.0)	56.7 (54.0-59.4)	55.3 (50.8-59.8)
Drug or alcohol treatment	48.1 (46.1-50.2)	42.1 (39.6-44.6)	38.7 (35.0-42.4)
Substance use disorder symptoms	79.7 (77.9-81.5)	73.9 (71.6-76.3)	72.2 (68.5-75.9)

Abbreviations: NA, not applicable; THC, tetrahydrocannabinol; UI, uncertainty interval.

^a^ In the neither applied group, only biological sex and census region were controlled by quota sampling; no weighting was applied. In the misclassification removal applied group, respondents showing careless patterns were excluded, with no weighting applied. In the misclassification removal and calibration applied group, both were applied.

^b^ Estimated population size not directly calculable from unweighted data.

^c^ Defined using the 7-item Generalized Anxiety Disorder scale.

^d^ Defined using the 9-item Patient Health Questionnaire.

^e^ Defined using the Short Warwick-Edinburgh Mental Well-being scale.

^f^ Other non-Hispanic race included respondents self-reporting American Indian or Alaska Native, Asian, Native Hawaiian or Other Pacific Islander, multiple non-Hispanic races, or other race.

**Figure 2.**
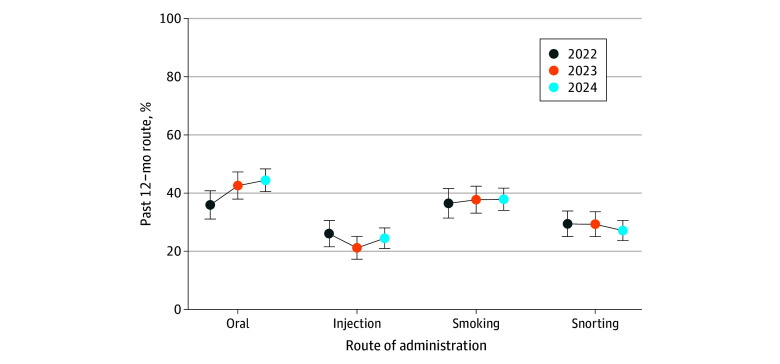
Route of Illicitly Manufactured Fentanyl Administration Over Time, 2022-2024 The proportion of adults who use illicitly manufactured fentanyl through oral routes has been increasing from 2022 to 2024 based on the bias-mitigated data. Error bars indicate 95% uncertainty intervals.

Using bias-mitigated data, oral use of IMF increased from 35.9% (95% UI, 31.1-40.7) in 2022 to 44.4% (95% UI, 40.3-48.5) in 2024 (Figure 2), which was the most used route of administration. Smoking, snorting, and injection use varied only a few percentage points across years. In 2024, smoking use was 37.9% (95% UI, 34.1-41.6), snorting use was 27.1% (95% UI, 23.5-30.7), and injection use was 24.5% (95% UI, 21.3-27.7).

## Discussion

Our bias-mitigated IMF estimates were notably higher than National Survey on Drug Use and Health estimates (in 2023, 0.8% vs 0.2%) and lower than other recent estimates (in 2024, 1.1% vs 7.5%).^3^ These 3 data sources triangulate the IMF use in the US. These data can, and should, be combined with others in multisource analyses qualitatively, as has been done with prescription opioids and psilocybin^11,12^ or in a statistically rigorous method to generate a combined estimate.^13^ These timely data show IMF use increased from 2023 to 2024, which is in contrast to recent declines in mortality.^14^ Our results suggest that increasing proportions of oral use, which has low bioavailability, may contribute to the divergence between use and mortality.^15^

Nondemographic composition mismatches and careless responses substantially influence estimates from nonprobability GPS. These results suggest adjustments have a cumulative effect on estimates, which are likely improved. Whether these methods successfully reduce bias may not be feasibly measured for all circumstances and was not directly measured for this IMF analysis. Calibration to benchmarks will correct for composition mismatch, but variables must be correlated with outcomes for adjustments to be effective and incomplete adjustment leads to incomplete composition bias mitigation.^5^ Using predefined calibration variables is appealing for surveillance designs but assumes that calibration sufficiently addresses composition bias across all outcomes. When detected perfectly, removing careless responses will reduce systematic error.^6^ But detection is imperfect, and some methods are more accurate than others. For example, self-report methods predicted a careless response phenotype at a lower rate than methods that analyzed response patterns.^8^ Multiple methods will more comprehensively detect careless responses than a single method.^6^ Methodologic variation in design can explain some differences between IMF estimates.

### Limitations

This study has limitations. This study avoids some, but not all, limitations of drug use GPS. Residual bias likely still exists.^13^ Positivity bias, where subgroups are not present in a sampling frame, is challenging to address. This survey excludes those without internet, institutionalized individuals, arrestees, and others who cannot enroll in online panels. The calibration method assumed benchmark estimates were accurate, and accuracy is poorly characterized for inherently stigmatized behaviors. Estimates should be interpreted in their context of an online, self-reporting panel, despite efforts to mitigate other forms of bias.

## Conclusions

Nonprobability GPSs offer many advantages as a tool for monitoring drug-related behavior. Bias mitigation should be multifaceted, addressing information, selection, and, where appropriate, confounding bias. These results suggest IMF use has shifted toward more oral use, which may contribute to observed lower mortality rates despite an increase in use.

## Notes

Supplement 1. **eMethods.** Additional Survey Design Information **eTable.** Recruitment Totals Per Launch

Supplement 2. Data Sharing Statement
