# Supplementary material for: Proactive Bias Mitigation When Using Online Survey Panels for Self-Reported Use of Illicitly Manufactured Fentanyl in the General Adult Population
Source: JAMA Health Forum. 2025 Nov 7;6(11):e254011. doi: 10.1001/jamahealthforum.2025.4011 (PMC12595535; doi:10.1001/jamahealthforum.2025.4011)
Supplement: Supplement 1. — eMethods. Additional Survey Design Information eTable. Recruitment Totals Per Launch [file jamahealthforum-e254011-s001.pdf]

Supplemental Online Content

Black JC, Rockhill KM, Schow N, Monte AA. Proactive bias mitigation when using online survey panels for self-reported use of illicitly manufactured fentanyl in the general adult population. *JAMA Health Forum*. 2025;6(11):e254011. doi:10.1001/jamahealthforum.2025.4011

eMethods. Additional Survey Design Information
eTable. Recruitment Totals Per Launch

This supplementary material has been provided by the authors to give readers additional information about their work.

© 2025 Black JC et al. *JAMA Health Forum*.

eMethods. Additional Survey Design Information

Two bias mitigation methods relevant to nonprobability GPS were implemented in NMURx: 1) exclusion of careless responses, and 2) calibration weighting. Respondents were excluded based on five criteria that measure diverse types of careless behaviors. Recruitment and exclusion totals are in eTable 2. Calibration weighting is a statistical process that matches marginal distributions of the sample to external benchmarks. Calibration adjustments were made for demographic (age, biologic sex, census region; benchmarked to the American Community Survey) and health-related metrics (tobacco use, limitations in daily activities; benchmarked to the National Health Interview Survey) to reduce composition bias that may not be addressed by quota sampling.

The EQUATOR CHERRIES guideline was followed for reporting from a closed online survey. Respondents are contacted via the panel company's portal. Individuals were presented an informed consent prior to initiating the survey. Personal information was not collected by the researchers. Each survey is tested by researchers prior to fielding. Adaptive questioning was used, so that respondents only saw questions relevant to the drugs they have used. For the illicitly manufactured fentanyl (IMF) question set, each respondent must first affirm they have used IMF in their lifetime, affirm they have used in the last 12 months, and indicate which route of administration they have used in the last 12 months (questions wording below). IMF is shown amongst other illicit drugs, and drug options are randomized to reduce order effect bias, with each drug appearing on the screen alone. Three scales validated for general population use measured anxiety (Generalized Anxiety Disorder Scale, 7-item), depression (Patient Health Questionnaire, 9-item), and wellbeing (Short Warwick-Edinburgh Mental Wellbeing Scale, 7-item). Race and ethnicity were self-reported in the same manner as US federal surveys, with two questions (one for Hispanic ethnicity and a check-all-that-apply for race). Anyone reporting Hispanic ethnicity was estimated separately from Non-Hispanic White, Non-Hispanic Black, and Non-Hispanic Other Race. Survey completeness was conducted for each question as it was submitted, and respondents were not allowed to change answers once submitted. Respondents were compensated via points, which could be redeemed for gift cards. Data from individuals who did not consent to be surveyed were not given to researchers to protect privacy, and therefore the view rates cannot be calculated. Participation and completion rates provided in eTable 1.

For the IMF questions, respondents are first shown this text and question:

"The question below is about substances that are made illegally (not by a drug company) and that are not obtained from a doctor. Have you ever used <DRUG>?" where <DRUG> is replaced with:

- "fentanyl not made by a drug company" or
- "drugs similar to fentanyl (carfentanil, acetylfentanyl, or furanylfentanyl)".

Slang terms were also provided.

Those who respond affirmatively were asked:

"When was the most recent time you used <DRUG>?" with response options of:

- Within the last week.
- 1 to 4 weeks ago.
- 1 to 3 months ago
- 4 to 12 months ago
- More than 12 months ago.

The first four options were combined for last 12-month use.

Those who reported use in the last 12 months were asked:

"In the last 12 months when you used <DRUG>, you…"

- swallowed it.
- crushed or chewed, and then swallowed it.
- dissolved it in your mouth. *(between cheek and gum, under tongue)*
- smoked it.
- snorted it.
- injected it. *(shot it up)*

The first three were combined into "oral" use.

© 2025 Black JC et al. *JAMA Health Forum*.

eTable. Recruitment Totals Per Launch

Recruitment Step	Spring 2022	Autumn 2022	Spring 2023	Autumn 2023	Spring 2024	Autumn 2024
Initiated Survey, n	40,589	43,518	49,400	60,286	42,521	43,731
Consented, n (Participation Rate, %)	39,527 (97.4%)	42,297 (97.2%)	48,207 (97.6%)	59,222 (98.2%)	41,540 (97.6%)	41,004 (93.8%)
Completed, n (Completion Rate, %)	30,834 (76.03%)	30,910 (71.0%)	36,075 (73.0%)	40,560 (67.3%)	30,834 (72.5%)	30,859 (70.6%)
Qualified, n[a]	30,004	30,003	29,999	29,928	30,003	30,002
Careless Response Excluded, n (% of qualified)	367 (1.2%)	599 (2.0%)	596 (2.0%)	803 (2.7%)	1,324 (4.4%)	962 (3.2%)
Final Sample Size	29,637	29,404	29,403	29,125	28,679	29,040

[a]Qualified participants were not part of a filled quota and were over the age of 18.

© 2025 Black JC et al. *JAMA Health Forum*.
