# Supplementary material for: Proactive Bias Mitigation When Using Online Survey Panels for Self-Reported Use of Illicitly Manufactured Fentanyl in the General Adult Population
Source: JAMA Health Forum. 2025 Nov 7;6(11):e254011. doi: 10.1001/jamahealthforum.2025.4011 (PMC12595535; doi:10.1001/jamahealthforum.2025.4011)
Supplement: Supplement 2. — Data Sharing Statement [file jamahealthforum-e254011-s002.pdf]

Data Sharing Statement

Black. Proactive Bias Mitigation When Using Online Survey Panels for Self-Reported Use of Illicitly Manufactured Fentanyl in the General Adult Population. *JAMA Health Forum*. Published November 07, 2025. doi:10.1001/jamahealthforum.2025.4011

Data
Data available: No
